# Nebulization versus standard application for topical anaesthesia during flexible bronchoscopy under moderate sedation – a randomized controlled trial

**DOI:** 10.1186/s12931-018-0926-5

**Published:** 2018-11-21

**Authors:** Tobias Müller, Christian Cornelissen, Michael Dreher

**Affiliations:** Department of Pneumology and Intensive Care Medicine, University Hospital RWTH Aachen, Pauwelsstraße 30, 52074 Aachen, Germany

**Keywords:** Lidocaine, Bronchoscopy, Anaesthesia, Nebulizers and vaporizers

## Abstract

**Background:**

Endobronchial administration of lidocaine is commonly used for cough suppression during diagnostic bronchoscopy. Recently, nebulization of lidocaine during bronchoscopies under deep sedation with fiberoptic intubation using a distinct spray catheter has been shown to have several advantages over conventional lidocaine administration via syringe. However, there are no data about this approach in bronchoscopies performed under moderate sedation. Therefore, this study compared the tolerability and safety of nebulized lidocaine with conventional lidocaine administration via syringe in patients undergoing bronchoscopy with moderate sedation.

**Methods:**

Patients requiring diagnostic bronchoscopy were randomly assigned to receive topical lidocaine either via syringe or via nebulizer. Endpoints were consumption of lidocaine and sedative drugs, as well as patient tolerance and safety.

**Results:**

Sixty patients were included in the study (*n* = 30 in each group). Patients required lower doses of endobronchial lidocaine when given via nebulizer versus syringe (164.7 ± 20.8 mg vs. 250.4 ± 42.38 mg; *p* < 0.0001) whereas no differences in the dosage of sedative drugs were observed between the two groups (all *p* > 0.05). Patients in the nebulizer group had higher mean oxygen saturation (96.19 ± 2.45% vs. 94.21 ± 3.02%; *p* = 0.0072) and a lower complication rate (0.3 ± 0.79 vs. 1.17 ± 1.62 per procedure; *p* = 0.0121) compared with those in the syringe group.

**Conclusions:**

Endobronchial lidocaine administration via nebulizer was well-tolerated during bronchoscopies under moderate sedation and was associated with reduced lidocaine consumption, a lower complication rate and better oxygenation compared with lidocaine administration via syringe.

**Trial registration:**

The study was registered with clinicaltrials.gov (NCT02262442; 13^th^ October 2014).

**Electronic supplementary material:**

The online version of this article (10.1186/s12931-018-0926-5) contains supplementary material, which is available to authorized users.

## Background

Flexible bronchoscopy is a procedure that is essential for the diagnostic work-up and management of patients with a variety of acute or chronic pulmonary diseases. The procedure is usually performed under sedation to increase patient comfort and tolerance, as suggested by current guidelines [1, 2]. Although there is no clear recommendation favouring one sedation regimen over any other, a combination of the short-acting benzodiazepine midazolam with opiates or propofol has been demonstrated to be effective and safe [1–4].

To reduce coughing and to keep the dosage of sedative drugs as low as possible, local anaesthetics such as lidocaine are administered topically to the upper airways and to the tracheobronchial tree through the working channel of the bronchoscope using a syringe [5]. However, this method may make it difficult to achieve even distribution of lidocaine in the bronchial system, resulting in incomplete anaesthesia of the airway walls. Therefore, spray catheters such as the Enk Fiberoptic Atomizer Set® have been developed, which allow nebulization of lidocaine during the bronchoscopy procedure using a constant oxygen flow [6]. This device was initially designed for use during awake fiberoptic intubation but can also be used safely during diagnostic bronchoscopies by respiratory physicians, as demonstrated in a recent clinical trial in which topical lidocaine administration via nebulizer was associated with reduced consumption of lidocaine and fentanyl compared with administration via syringe [7, 8]. However, because all diagnostic bronchoscopies in that trial were performed under deep sedation to allow fiberoptic intubation, the conclusions drawn cannot necessarily be transferred to procedures performed under moderate or light sedation without fiberoptic intubation. Therefore, the aim of the current study was to investigate whether the use of the nebulizer system for lidocaine delivery during flexible bronchoscopy is superior to conventional lidocaine application via syringe when the procedure is performed under moderate sedation.

## Methods

The study protocol was approved by the Institutional Review Board for Human Studies at RWTH University, Aachen, Germany (14–074), and was performed in accordance with the ethical standards laid down in the Declaration of Helsinki. Written informed consent was obtained from all patients prior to inclusion into the study. The study was registered with clinicaltrials.gov (NCT02262442; 13th October 2014), the full trial protocol is accessible on request.

### Patients

From the 20th October 2014 until the 29th November 2017 patients requiring diagnostic bronchoscopy at the university hospital RWTH Aachen were consecutively included in the study. Diagnostic procedures such as broncho-alveolar lavage, endobronchial or transbronchial biopsies, or brush cytology were permitted. However, we did not include patients requiring cryobiopsy or endobronchial ultrasound because these procedures are performed under deep sedation including fiberoptic intubation in our institution. Exclusion criteria were epilepsy, severe neurological or psychiatric disorder, hemodynamic instability requiring catecholamine treatment, decompensated heart failure, severe respiratory failure (pH < 7.35, arterial oxygen pressure [PaO_2_] < 55 mmHg despite supplemental oxygen), history of upper airway surgery or radiation, allergy to lidocaine, propofol or midazolam, or bleeding disorder. Standard laboratory tests (blood cell count; coagulation) and pulmonary function tests were performed prior to inclusion into the study.

### Study design

For baseline measurements, arterial blood gas (ABG) analyses (ABL 800 flex, Radiometer, Copenhagen, Denmark) were performed using the arterialized earlobes of all patients while breathing room air without supplemental oxygen. Oxygen saturation (SpO_2_) and heart rate were continuously monitored and recorded every 5 min from the beginning of the intervention until an ALDRETE (global assessment of post-aesthetic condition) score of at least 9 was recorded after the intervention. [8, 9] Another ABG measurement was performed after completion of the procedure. All complications occurring during the procedure and within 24 h were recorded. Complications were defined as drops SpO_2_ < 90%, need for short-term ventilation during the procedure, endobronchial bleedings, hypotension, pneumothorax, post-interventional admission to an intermediate or intensive care unit, or other events judged as complication by the investigator.

### Sedation and bronchoscopy

All procedures were performed by the same two experienced investigators (first and last authors). Patients were randomized to lidocaine application via syringe (syringe group) or via nebulizer (nebulizer group) by our clinical trial centre using the sealed envelope system. All patients were unaware of treatment group allocation. Nebulization of lidocaine was performed using the Enk Fiberoptic Atomizer Set® (Cook Medical, Bloomington, USA) with an oxygen flow of 10 L/min, as suggested by the manufacturer. Monitoring included electrocardiogram, SpO_2_, pre- and post-interventional ABG analysis and non-invasive blood pressure (NIBP). Before starting bronchoscopy, patients received supplemental oxygen through a nasal cannula at a flow rate of ≥2 L/min, adjusted to maintain SpO_2_ at > 95%. The oxygen flow rate was recorded throughout the entire protocol. The sedation regimen was similar as described in our previous study. [8] However, a target sedation level of moderate was sought and – in contrast to the study mentioned above – patients were not intubated for the procedure. [8] Briefly, all patients received an intravenous bolus injection of midazolam. After a waiting period, propofol boli were administered at the investigator’s discretion until sufficient patient tolerance for the procedure was achieved. If considered necessary bolus doses of fentanyl were permitted, too. Lidocaine (20 mg/mL) was administered during the bronchoscopy either by nebulizer or by syringe as per randomization. Target zones for lidocaine administration in both groups were the vocal cords, the trachea, the main carina and the main bronchi. Typically, 20 to 40 mg of lidocaine were injected at each site depending on patient tolerance. There was only a short waiting period of about 10s between lidocaine administration at the different sites in both groups. Lidocaine administration at these sites was followed by inspection of the airways without any delay. Additional injections of lidocaine were at the discretion of the bronchoscopists, e. g. if excessive coughing occurred.

### Patient tolerance

Patient tolerance was assessed using the Global Tolerance Score, based on a visual analogue scale (VAS; 0 = no bother, 100 = intolerable); the same VAS was used to rate four specific sensations: nausea, asphyxia, cough and pain (0 = non-existent, 100 = unbearable). The Tolerance Score, defined as the arithmetic mean of global tolerance VAS score and the mean of scores for the 4 specific sensations, was calculated as described previously [4, 8]. The American Society of Anaesthesiologists’ (ASA) score was used to assess physical status [10]. The ALDRETE score was used to assess recovery after bronchoscopy [9].

### Study endpoints

Primary endpoints were the dosages of administered propofol and lidocaine. Secondary endpoints included the dosages of midazolam and fentanyl, post procedural blood gas values, the duration of the bronchoscopy, the occurrence of complications and the time span until an ALDRETE score of at least 9 was recorded after the procedure.

### Statistical analysis

Statistical analysis was performed using GraphPadPrism (GraphPad Software, La Jolla, USA). Unless otherwise stated, all data are presented as mean ± standard deviation (SD) after testing for normal distribution (Kolmogorov-Smirnov test). Pre- and post-interventional measurements were compared using the paired t-test for normally distributed data. A two-group comparison was performed using the unpaired t-test for normally distributed data. For normally distributed data, the 95% confidence interval of the mean (95% CI) is given where appropriate. For non-normally distributed data, the Wilcoxon signed rank test was used and the interquartile range is given. The Fisher’s exact test was used for categorical data. Statistical significance was defined as a *p* value < 0.05.

## Results

### Patients

A total of 60 patients (30 in each group) were included in the study. The nebulizer and syringe groups were comparable at baseline, with similar ABG, pulmonary function, smoking status and ASA classification (Table 1). Indications for bronchoscopy and the diagnostic interventions performed during bronchoscopy are summarized in Table 2. The mean duration of the procedure was similar and not significantly different between the two groups (syringe group: 14.67 ± 5.44 min vs. nebulizer group: 12.17 ± 4.6 min; Δ 2.5 ± 1.3; 95% CI –0.10, 5.1; *p* = 0.0594).

**Table 1:** Patient demographic data, lung function parameters and blood gas analysis (room air breathing) at baseline

	Lidocaine		Difference between means (95% CI)	*p*-value
	Syringe (*n* = 30)	Nebulizer (*n* = 30)		
Male, n (%)	18 (60)	21 (63.6)	–	0.8001^*^
Age, years	64.47 ± 11.56	67.9 ± 9.08	−3.43 ± 2.68 (− 8.81, 1.94)	0.2059^#^
Body weight, kg	79.33 ± 16.5	86.64 ± 15.81	−7.31 ± 4.17 (− 15.66, 1.05)	0.0852^#^
Smoking history, pack years	5 (0–30)	5 (0–30)	–	0.7064^§^
FEV_1_, % predicted	77.5 ± 21.1	74.18 ± 21.24	3.32 ± 5.66 (−8.03, 14.68)	0.5600^#^
PaO_2_, mmHg	69.29 ± 18.6	66.56 ± 12.99	2.73 ± 4.14 (−5.56, 11.02)	0.5124^#^
PaCO_2_, mmHg	38.1 ± 8.84	36.36 ± 3.64	1.74 ± 1.75 (−1.75, 5.23)	0.3288^#^
ASA 1/2 or 3/4, n (%)	16 (53.3); 14 (46.7)	16 (53.3)	–	> 0.9999^*^

Data are presented as mean ± standard deviation, median (interquartile range) or number of patients (%)

*Fisher’s exact test. ^#^Unpaired t-test. ^§^Mann Whitney test

ASA, American Society of Anesthesiologists; CI, confidence interval; FEV_1_, forced expiratory volume in 1 s; PaCO_2_, arterial partial pressure of carbon dioxide; PaO_2_, arterial partial pressure of oxygen

**Table 2:** Indications for bronchoscopy and type of diagnostic intervention during the procedure

	Lidocaine	
	Syringe (*n* = 30)	Nebulizer (*n* = 30)
Indications for bronchoscopy, n (%)		
Lung cancer	7 (23.3)	12 (40)
Interstitial lung disease	12 (40)	5 (16.6)
Unexplained pulmonary opacities	6 (20)	4 (13.3)
Hemoptysis	2 (6.7)	3 (10)
Other	3 (10)	6 (20)
Diagnostic interventions, n (%)		
Inspection only	5 (16.6)	10 (33.3)
Broncho-alveolar lavage	20 (66.6)	16 (53.3)
Endobronchial biopsy	4 (13.3)	2 (6.7)
Transbronchial biopsy	5 (16.6)	3 (10)
Bronchial brushing	0 (0)	1 (3.3)

### Medication

**Fig. 1:**
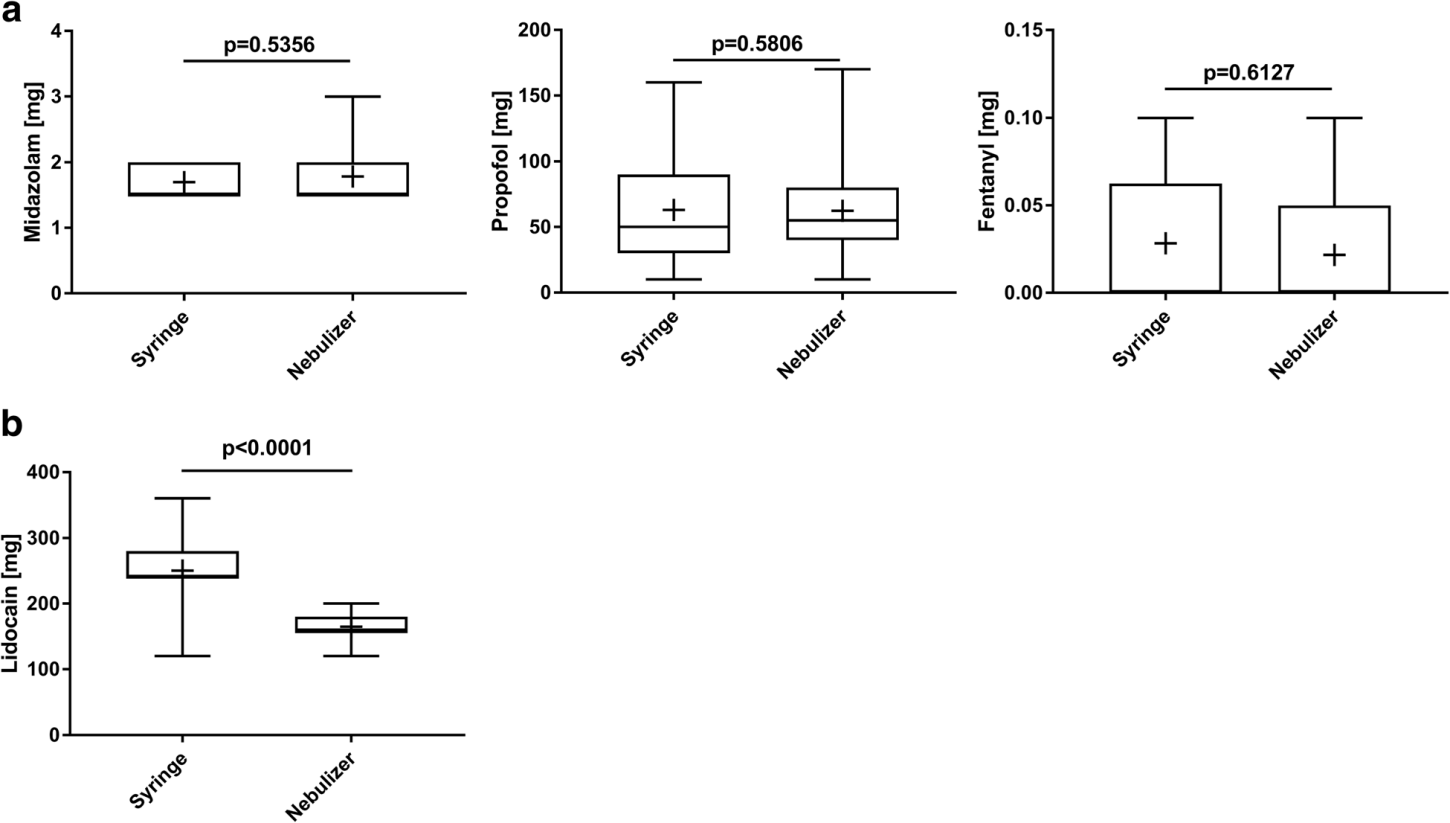
Dosages of administered sedative drugs and lidocaine. Median doses of (**a**) intravenously administered midazolam (right), propofol (middle), and fentanyl (right), and (**b**) intrabronchial lidocaine. Boxes represent the interquartile range, whiskers show maximum and minimum, and + indicates mean value

All patients received an intravenous bolus of midazolam before the start of bronchoscopy. No additional bolus doses of midazolam were given during the procedure. The dosage of midazolam was similar in the two groups (syringe group: 1.7 ± 0.25 mg vs. nebulizer group: 1.78 ± 0.36 mg; *p* = 0.5356) (Fig 1a, left). There was no significant between-group difference in the amount of propofol administered as boli (syringe group: median 63 ± 40.36 mg vs. nebulizer group: 62.33 ± 33.6 mg; *p* = 0.5806) (Fig 1a, middle). A similar proportion of patients in both groups received additional bolus doses of fentanyl (10 vs. 8 patients), resulting in similar fentanyl dosages (syringe group: 0.0283 ± 0.0429 mg vs. nebulizer group: 0.0217 ± 0.0387 mg; *p* = 0.6127) (Fig 1a, right). Doses of intrabronchial lidocaine were higher in the syringe versus nebulizer group (250.4 ± 42.38 mg vs. 164.7 ± 20.8 mg, respectively; *p* < 0.0001) (Fig 1b).

### Vital signs and oxygen flow rate during bronchoscopy

Mean oxygen saturation was significantly higher in the nebulizer compared with the syringe group (*p* = 0.0072) (Table 3). Interestingly, the mean oxygen flow rate needed to maintain SpO_2_ at > 95% was significantly higher in the syringe versus nebulizer group (*p* = 0.0002) (Table 3). No other between-group differences in vital signs were observed (all *p* > 0.05) (Table 3).

**Table 3:** Vital signs during bronchoscopy

	Lidocaine		Difference between means (95% CI)	*p*-value
	Syringe (*n* = 30)	Nebulizer (*n* = 30)		
Heart rate, beats/min	85.4 ± 10.06	79.84 ± 15.31	5.56 ± 3.34 (−1.13, 12.25)	0.1018^#^
SBP, mmHg	125.2 ± 14.19	125.2 ± 21.55	0.06 ± 4.71 (−9.37, 9.49]	0.9894^#^
DBP, mmHg	71.7 ± 10.47	69.42 ± 12.56	2.27 ± 2.99 (−3.7, 8.25)	0.4498^#^
SpO_2_, %	94.21 ± 3.02	96.19 ± 2.45	−1.98 ± 0.71 (− 3.4, −0.56)	0.0072^#^
Oxygen flow rate, L/min	2.8 (2–3.33)	2 (2–2)	–	0.0002^§^

Data are presented as mean ± standard deviation or median (interquartile range)

^#^Unpaired t-test. ^§^Mann Whitney test

DBP, diastolic blood pressure; SBP, systolic blood pressure; SpO_2_, oxygen saturation

### Arterial blood gas analysis

**Fig. 2:**
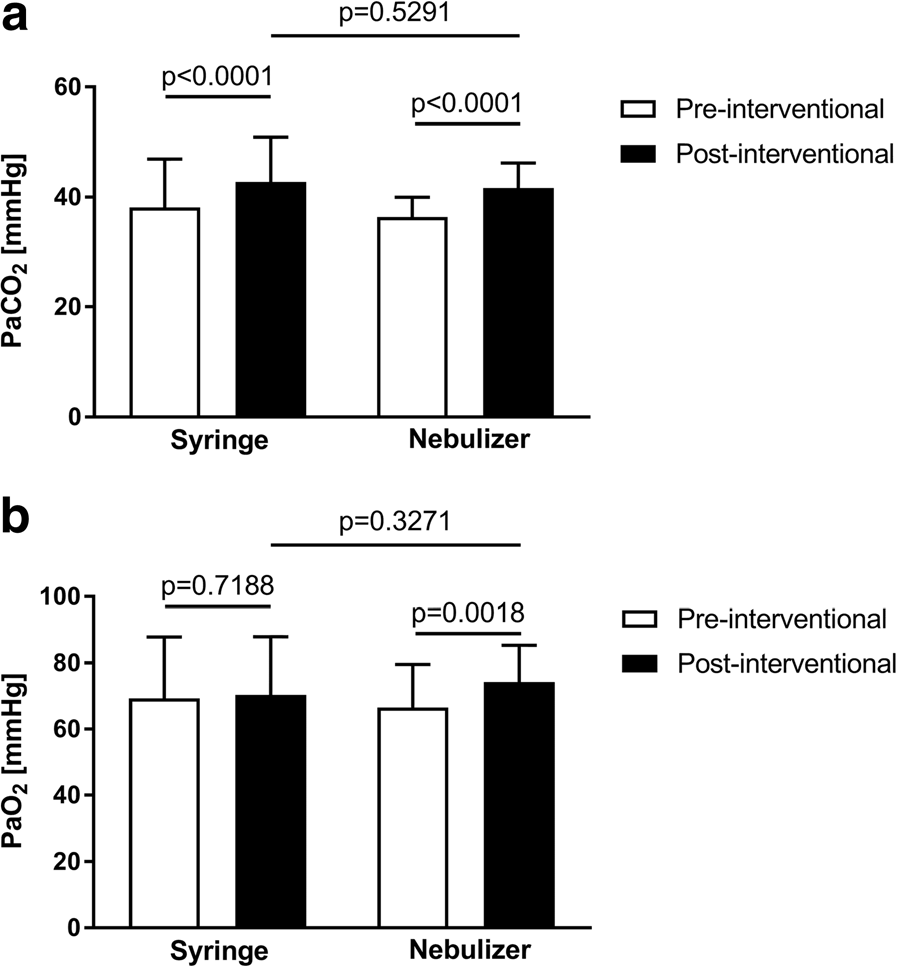
Pre- and postinterventional arterial blood gas analysis. Arterial pressure of (**a**) carbon dioxide (PaCO_2_) and (**b**) oxygen (PaO_2_) before and after bronchoscopy. Data are given as mean + standard deviation

Compared with pre-procedural values, post-procedural partial pressure of carbon dioxide in arterial blood (PaCO_2_) was significantly higher in both groups (syringe: 42.75 ± 8.18 mmHg vs. 38.1 ± 8.84 mmHg; *p* < 0.0001; nebulizer: 41.67 ± 4.57 mmHg vs. 36.36 ± 3.64 mmHg; *p* <  0.0001) whereas there were no significant post-procedural differences between the two groups in PaCO_2_ (Δ 1.1 ± 1.7 mmHg; 95% CI –2.3, 4.5 mmHg; *p* = 0.5291) or in the difference between pre- and post-procedural PaCO_2_ between the two groups (Δ − 07 ± 1.0 mmHg; 95% CI –2.6, 1.3 mmHg; *p* = 0.5101) (Fig. 2a).

Post-procedural PaO_2_ values did not differ significantly between the 2 groups (syringe: 70.4 ± 17.5 mmHg vs. nebulizer: 74.2 ± 11.2 mmHg; *p* = 0.3271). However, a significant increase between pre- and post-interventional PaO_2_ was seen in the nebulizer group (Δ 7.6 ± 12.1 mmHg; 95% CI 3.1, 12.1 mmHg; *p* = 0.0018) but not in the syringe group (Δ 1.1 ± 16.8 mmHg; 95% CI –5.2, 7.4 mmHg; *p* = 0.7188) (Fig 2b).

### Complications and recovery

The proportion of procedures with at least one complication was higher in the syringe group compared with the nebulizer group, although this did not reach statistical significance (syringe: *n* = 14 [46.67%] vs. nebulizer: *n* = 6 [20%]; *p* = 0.0539). However, the complication rate per procedure was significantly higher in the syringe group (1.17 ± 1.62 vs. nebulizer: 0.3 ± 0.79; *p* = 0.0121), almost exclusively due to more episodes of SpO_2_ < 90% (syringe 1.03 ± 1.33 per procedure vs. nebulizer: 0.27 ± 0.78 per procedure; *p* = 0.0070). Other complications were very rare and not significantly different between the groups (syringe group: 0.13 ± 0.57 per procedure vs. nebulizer: 0.03 ± 0.18 per procedure; *p* = 0.7458).

There was no between-group difference in the mean time required to reach an ALDRETE score of at least 9 (syringe: 4 ± 3.6 min vs. nebulizer: 4.7 ± 4.1 min; Δ − 0.7 ± 1.0 min; 95% CI –2.7, 1.3 min; *p* = 0.5068).

### Tolerance scores

**Fig. 3:**
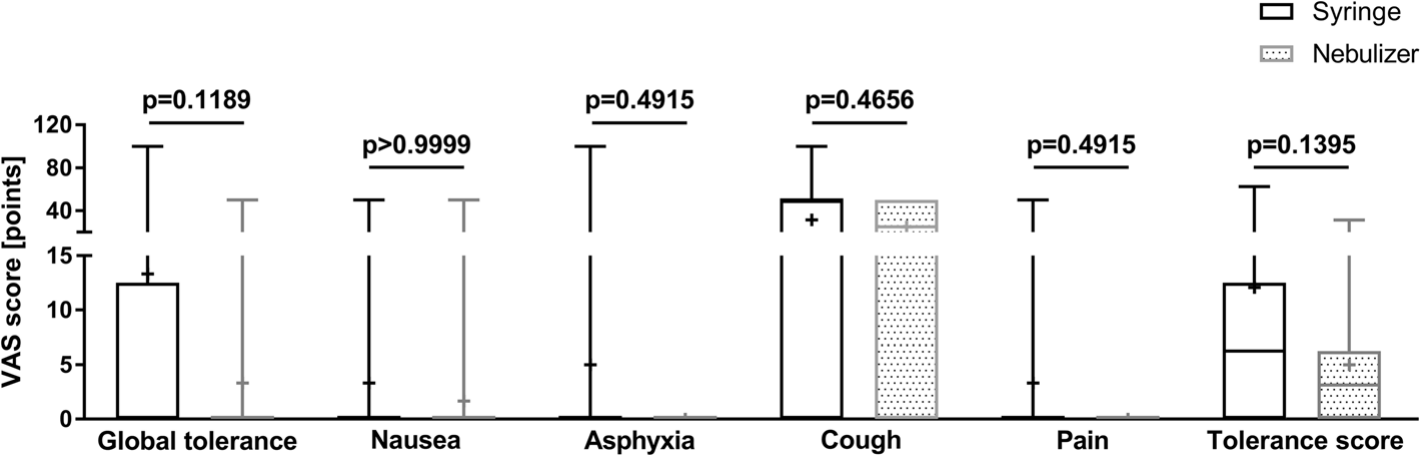
Tolerance Scores. Median visual analogue scale (VAS) scores for global tolerance, nausea, asphyxia, cough and pain (0 = non-existent; 100 = unbearable). Boxes represent the interquartile range, whiskers report maximum and minimum, and + indicated mean value

VAS scores for global tolerance, nausea, asphyxia, cough and pain were not statistically different between the two groups (Fig 3). The tolerance score tended to be higher in the syringe versus nebulizer group, but the difference was not statistically significant (syringe: 12.08 ± 17.21 points vs. nebulizer: 5 ± 7.94 points; *p* = 0.1395) (Fig 3).

## Discussion

The current study compared two different methods of intrabronchial lidocaine administration during bronchoscopy under moderate sedation. The results showed that administration via nebulizer was associated with reduced consumption of lidocaine, better oxygenation during the procedure and a favourable safety profile compared to lidocaine administration via syringe.

To facilitate the procedure and to increase patient tolerance, comfort, and cooperation, most centres perform flexible bronchoscopy under sedation in accordance with current guidelines [1, 2]. Previous studies demonstrated that sedation with two or even three different drugs is safe and might have several advantages over sedation with just one drug [3, 4, 11, 12]. Procedures in the present study were performed under light to moderate sedation, hence a regimen consisting of midazolam induction followed by propofol bolus doses and as needed fentanyl bolus doses was chosen. There was no difference between the nebulizer and the syringe group with respect to the amount of midazolam, propofol, or fentanyl administered. This is in contrast to our previous study in which less fentanyl was needed in the nebulizer versus syringe group, possibly due to better cough suppression [8]. However, in that study a deeper level of sedation was needed because all patients underwent fiberoptic intubation under maintenance of spontaneous breathing resulting in a markedly longer procedure duration [8]. The findings of both studies together could suggest that more effective cough suppression with nebulized versus syringe lidocaine might be less important during shorter bronchoscopies requiring only moderate sedation.

Topical lidocaine administration during flexible bronchoscopy is widely used and is recommended by current guidelines [1, 2]. Nevertheless, there is still concern about the side effects of endobronchial lidocaine, including cardiac arrhythmias, seizures or deterioration in pulmonary function [13–15]. Therefore, the dosage of lidocaine should be kept as low as possible. Our results show that lidocaine dosages during diagnostic fiberoptic bronchoscopy can be reduced by administering the drug via nebulizer without increasing the dosage of sedative drugs. This is consistent with our previous findings showing reduced lidocaine consumption during bronchoscopy under deep sedation when nebulized lidocaine was used [8]. In that study it is important to note that the reduced dosage of endobronchial lidocaine in the nebulizer group did not reduce patient comfort or lead to excessive coughing. Similar observations have been reported during fiberoptic intubation by anaesthesiologists, where lidocaine administered via nebulizer resulted in better cough suppression compared with lidocaine administered via syringe [16, 17]. One possible explanation for this observation might be better distribution of lidocaine on the surface of the mucosa in the upper airways and the tracheobronchial system, resulting in increased airway anaesthesia. However, apart from a better diffusion of lidocaine in the tracheobronchial system anaesthesia of the airway wall also depends on additional factors, e. g. preventive versus as needed lidocaine administration, the locations of lidocaine deposition, or the time span between lidocaine administration and the passage of the bronchoscope. As lidocaine was administered preventively, at the same sites and there was no delay between lidocaine administration and the beginning of the bronchoscopy in the nebulizer and the syringe group, variations in these factors were kept as low as possible, though a certain bias cannot be excluded.

Mean SpO_2_ was higher whereas the supplemental oxygen flow rate needed to maintain SpO_2_ > 95% was lower in the nebulizer compared with the syringe group. Furthermore, arterial post-interventional PaO_2_ versus pre-interventional PaO_2_ increased significantly in the nebulizer but not in the syringe group. Given that the dosages of sedative drugs, which can possibly cause respiratory depression, were similar in both groups this effect can most likely be attributed to the constant oxygen flow used for the nebulization of lidocaine, in accordance with previous studies [8, 16]. Improved oxygenation during bronchoscopy should be of clinical relevance, especially for patients with pre-existing respiratory failure or sleep apnoea. Therefore, the improvement in oxygenation is another potential advantage of administrating lidocaine via nebulizer. In addition, the complication rate was lower in the nebulizer group, largely due to a reduced number of times when SpO_2_ was < 90%. Again, this observation can be explained by better oxygenation due to the use of a constant oxygen flow for the nebulization of lidocaine [8, 16].

Consistent with existing literature, we did not find any statistically significant differences in patient tolerance between the nebulizer and the syringe groups, although our previous data indicated a trend towards better tolerance when the nebulizer is used [8]. In addition, patients recovered quickly after the procedure and no between-group differences were observed, consistent with the similar amounts of sedative drugs administered in both groups.

There was a slight imbalance between the groups in terms of diagnostic interventions as bronchoscopies without sampling were more common in the nebulizer group which must be considered as a limitation of our study. Nevertheless, this imbalance was not statistically significant and did neither increase the duration of the procedure nor the dosage of sedative drugs.

## Conclusions

In summary, administration of topical lidocaine via nebulizer during flexible bronchoscopy under moderate sedation is associated with reduced consumption of lidocaine compared with standard administration via syringe. Furthermore, nebulizing lidocaine during bronchoscopy was associated with improved oxygenation during the procedure and fewer peri-interventional complications. Therefore, nebulizers can be recommended for usage during diagnostic bronchoscopy, especially for patients suffering from respiratory failure.

## Additional files

Additional file 1: Supplementary data. (XLSX 20 kb)

## Abbreviation

ABG: Arterial blood gas
